# DI/LC–MS/MS-Based Metabolome Analysis of Plasma Reveals the Effects of Sequestering Agents on the Metabolic Status of Dairy Cows Challenged with Aflatoxin B_1_

**DOI:** 10.3390/toxins11120693

**Published:** 2019-11-26

**Authors:** Ibukun Ogunade, Yun Jiang, Andres Pech Cervantes

**Affiliations:** 1 College of Agriculture, Communities, and the Environment, Kentucky State University, Frankfort, KY 40601, USA; 2 Department of Animal Sciences, University of Florida, Gainesville, FL 32611, USA; ogunadeibukun@gmail.com; 3 Agricultural Research Station, Fort Valley State University, Fort Valley, GA 31030, USA; xtiemira@gmail.com

**Keywords:** aflatoxin, biomarker, dairy cows

## Abstract

The study applied a targeted metabolomics approach that uses a direct injection and tandem mass spectrometry (DI–MS/MS) coupled with a liquid chromatography–tandem mass spectrometry (LC–MS/MS)-based metabolomics of plasma to evaluate the effects of supplementing clay with or without *Saccharomyces cerevisiae* fermentation product (SCFP) on the metabolic status of dairy cows challenged with aflatoxin B_1_. Eight healthy, lactating, multiparous Holstein cows in early lactation (64 ± 11 DIM) were randomly assigned to one of four treatments in a balanced 4 × 4 duplicated Latin square design with four 33 d periods. Treatments were control, toxin (T; 1725 µg aflatoxin B_1_ (AFB_1_)/head/day), T with clay (CL; 200 g/head/day), and CL with SCFP (YEA; 35 g of SCFP/head/day). Cows in T, CL, and YEA were dosed with aflatoxin B_1_ (AFB_1_) from days 26 to 30. The sequestering agents were top-dressed from day 1 to 33. On day 30 of each period, 15 mL of blood was taken from the coccygeal vessels and plasma samples were obtained from blood by centrifugation and analyzed for metabolites using a kit that combines DI–MS/MS with LC–MS/MS-based metabolomics. The data were analyzed using the GLIMMIX procedure of SAS. The model included the effects of treatment, period, and random effects of cow and square. Significance was declared at *p* ≤ 0.05. Biomarker profiles for aflatoxin ingestion in dairy cows fed no sequestering agents were determined using receiver–operator characteristic (ROC) curves, as calculated by the ROCCET web server. A total of 127 metabolites such as amino acids, biogenic amines, acylcarnitines, glycerophospholipids, and organic acids were quantified. Compared with the control, T decreased (*p* < 0.05) plasma concentrations of alanine, leucine, and arginine and tended to decrease that of citrulline. Treatment with CL had no effects on any of the metabolites relative to the control but increased (*p* ≤ 0.05) concentrations of alanine, leucine, arginine, and that of citrulline (*p* = 0.07) relative to T. Treatment with YEA resulted in greater (*p* ≤ 0.05) concentrations of aspartic acid and lysine relative to the control and the highest (*p* ≤ 0.05) plasma concentrations of alanine, valine, proline, threonine, leucine, isoleucine, glutamic acid, phenylalanine, and arginine compared with other treatments. The results of ROC analysis between C and T groups revealed that the combination of arginine, alanine, methylhistidine, and citrulline had sufficient specificity and sensitivity (area under the curve = 0.986) to be excellent potential biomarkers of aflatoxin ingestion in dairy cows fed no sequestering agents. This study confirmed the protective effects of sequestering agents in dairy cows challenged with aflatoxin B_1_.

## 1. Introduction

Fungal spoilage of livestock feeds continues to be a major problem for feed security because of reduced palatability and loss of nutritive value [1,2]. Worse still, the affected commodity may be contaminated with toxic fungal secondary metabolites such as mycotoxins [3]. Aflatoxin B1 is the most studied among the fungal secondary metabolites because it is carcinogenic and poses a serious public health issue due to its transfer from diet to animal products such as milk, meat, and eggs [4,5]. Consequently, most studies have focused on the use of sequestering agents such as clay and Saccharomyces cerevisiae-based additives to counteract the negative effects of aflatoxin on performance and reduce its transfer to animal products [6]. Most of these studies have evaluated the effects of aflatoxins with or without sequestering agents on the metabolic status of ruminants using few biochemical parameters such as plasma liver enzymes and blood cell counts [6,7], which offer very little in terms of metabolic inferences [8]. A comprehensive analysis of the metabolic profile of animals to aflatoxin exposure is needed to reveal a robust metabolic inference and identify biomarkers of aflatoxin ingestion, which may allow early detection of aflatoxicosis, poisoning caused by ingesting aflatoxins, in livestock.

In recent years, metabolomics has been extensively used in basic and applied research to study metabolic processes and identify biomarkers responsible for metabolic characteristics [9]. Metabolomics can reveal the metabolic response of a biological system to several factors including stress, environmental alterations, and dietary change [10,11]. Previously, our group applied high-resolution proton nuclear magnetic resonance spectroscopy (1H NMR)-based metabolomics of plasma to identify plasma metabolites such as acetic acid, arginine, ethanol, alanine, methylhistidine, and proline as biomarkers of aflatoxin ingestion in dairy cows fed no sequestering agents [12]. However, liquid chromatography coupled to tandem mass spectrometry (LC–MS/MS) offers several advantages including high sensitivity and specificity and the potential to analyze disease-associated metabolic changes and quantify hundreds of metabolites in one run [13,14]. Thus, the objective of this study was to evaluate the effects of supplementing clay with or without *Saccharomyces cerevisiae* fermentation product (SCFP) on the plasma metabolomics profile of dairy cows challenged with aflatoxin B_1_ using a combination of direct injection and tandem mass spectrometry (DI–MS/MS) with a reverse-phase LC–MS/MS.

## 2. Results and Discussions

**Figure 1:**
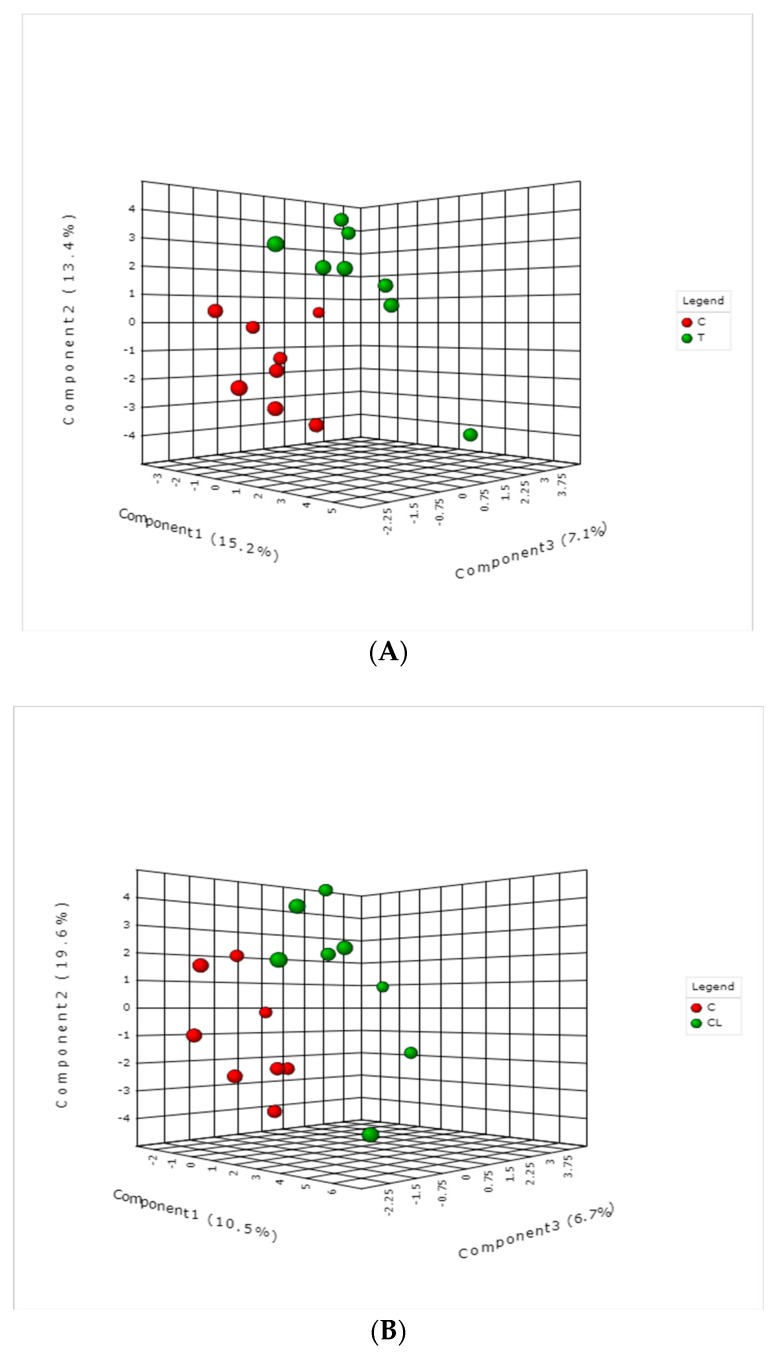

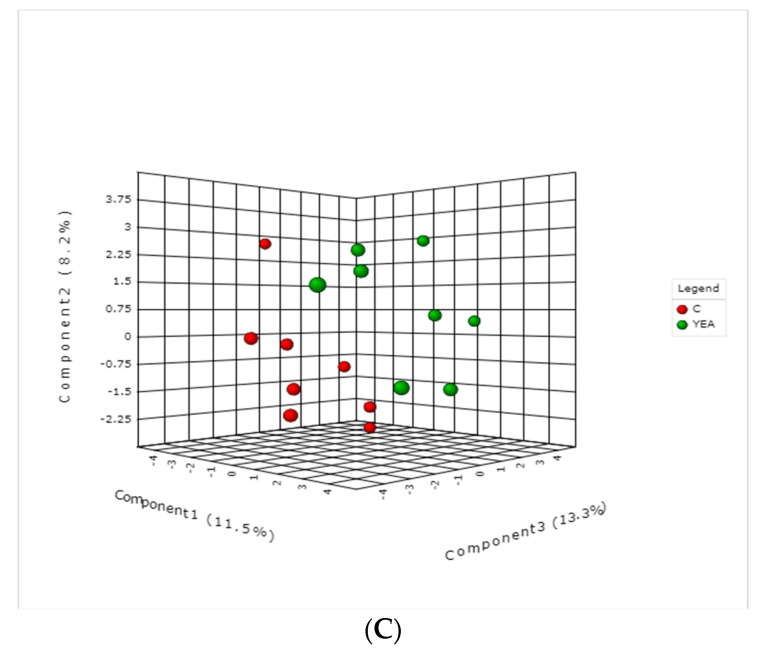
Partial least squares discriminant analysis score plots of (**A**) control vs. toxin groups, (**B**) control vs. clay groups, and (**C**) control vs. clay + *S. cerevisiae* fermentation product (YEA) groups.

A total number of 127 metabolites belonging to groups such as biogenic amines, acylcarnitines, amino acids, glycerophospholipids, monosaccharides, organic acids, and hexoses were identified and quantified (Table S1). The partial least squares discriminant analysis modelling (Figure 1) revealed slight separations between the control and each of T (toxin), CL (clay), and YEA (CL with SCFP) groups, indicating that the dietary treatment altered the plasma metabolome of the dairy cows. 

**Figure 2:**
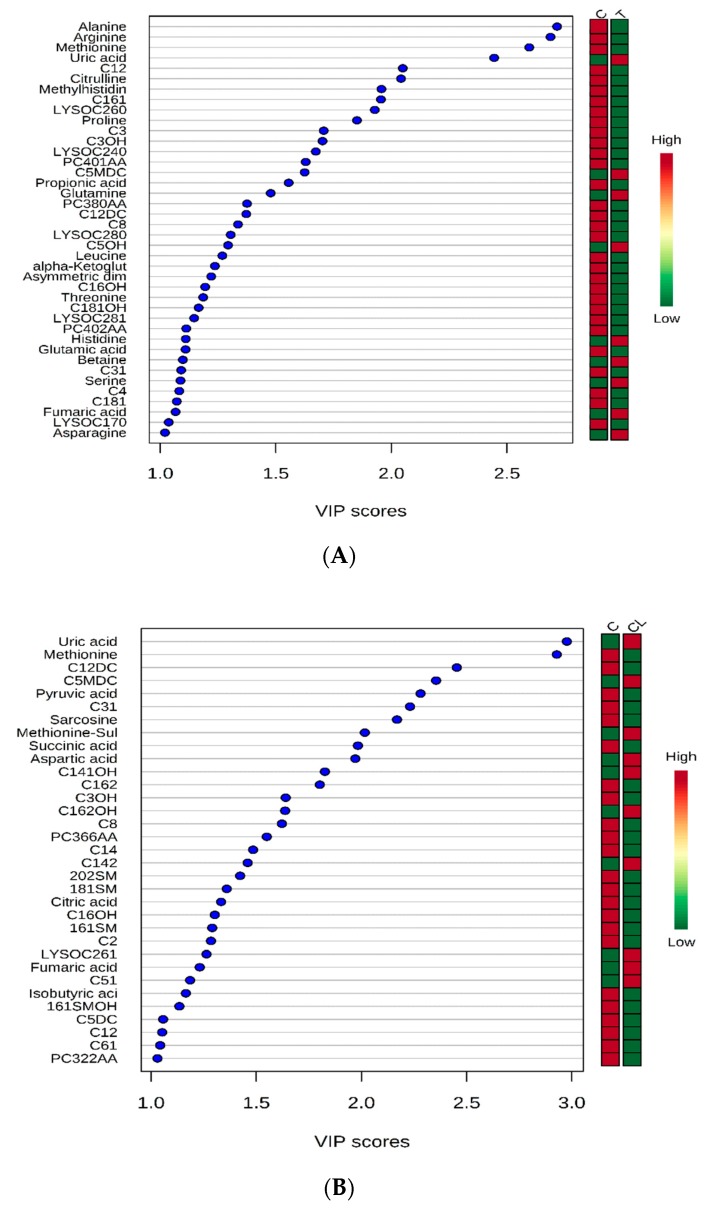

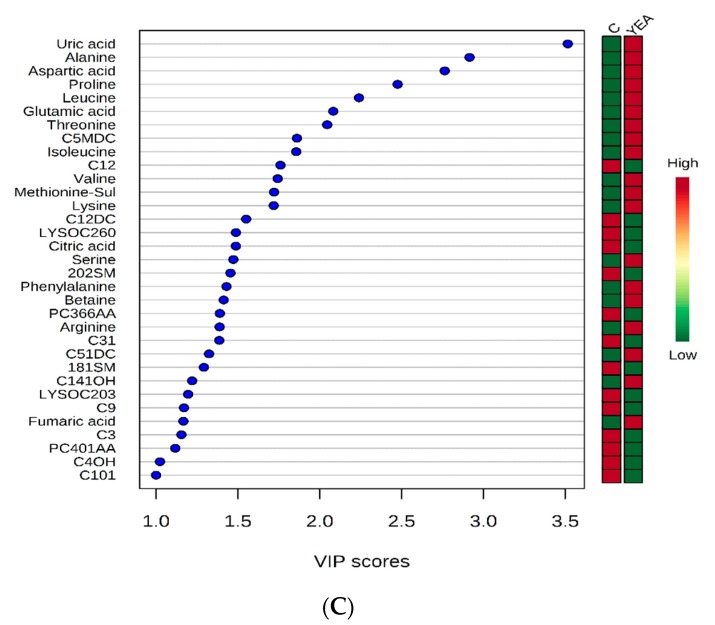
Variables ranked by variable importance in projection (VIP) between control and toxin groups (**A**), control and clay groups (**B**), and control and clay + *S. cerevisiae* fermentation product groups (**C**). Acylcarnitines: C12, C161, C3, C3OH, C12DC, C8, C5OH, C16OH, C181OH, C31, C4, C181, C12DC, C5MDC, C31, C141OH, C162, C3OH, C162OH, C8, C14, C142, C16OH, C2, C51, C5DC, C12, C61, C101, C4OH, C3, C9, C31, C12DC, C5MDC. Glycerophospholipids: LYSOC260, LYSOC240, PC401AA, LYSOC280, LYSOC281, PC402AA, LYSOC170, 202SM, 181SM, 161SM, LYSOC261, 161SMOH, PC322AA, PC401AA, LYSOC203.

The ranking of the metabolites by variable importance in projection (VIP) >1 showed that 40, 33, and 33 metabolites contributed to respective separations between control and each of T, CL, and YEA groups, respectively (Figure 2).

When the metabolites with VIP >1 were statistically analyzed based on the design of this experiment, the concentrations of 13 metabolites were affected by dietary treatment. Relative to the control, T diet decreased (*p* < 0.05) the plasma concentrations of alanine, leucine, and arginine and tended to decrease (*p* = 0.07) that of citrulline (Table 1). These results agree with our earlier study that showed a similar trend using 1H-NMR [12]. This study also agrees with a recent study that reported reduced concentrations of some amino acids such as leucine, isoleucine, valine, and phenylalanine in dairy cows exposed to 40 µg/kg aflatoxin B_1_ (AFB1) for 7 days [8].

Aflatoxins are known to impair protein formation by interfering with enzymes and substrates required for processes such as initiation, transcription, and translation that are involved in protein synthesis [15]. Pate et al. [16] reported reduced expression of mechanistic target of rapamycin (mTOR), a major regulator of protein synthesis in the tissues of mammals [17], in the liver of dairy cows fed 100 μg of AFB1/kg of dietary DMI for 3 days. This is probably a consequence of reduced plasma concentrations of leucine, which is known to activate the mTOR signaling pathway [18]. Mechanistic target of rapamycin plays a major role in regulating innate and adaptive immune responses in animals and humans because antibodies, interferons, and other immune cells are made up of proteins [18]. Arginine and its precursor, citrulline, play a vital role in regulating immune response during inflammatory stress because arginine serves as a sole precursor for synthesis of immune modulators such as polyamines, proline, and agmatine [19]. Taken together, this explains observed immunosuppression in animals exposed to AFB_1_ in several studies [20,21]. Alanine is the major glucogenic amino acid vital for glucose metabolism in ruminants [22]. However, reduced concentration of alanine observed in this study is not an evidence that carbohydrate metabolism was affected by AFB_1_ because plasma pyruvate and glucose concentrations were not affected. It is important to note that the concentration of AFB1 dosed (75 μg/kg) in this study exceeded that of the FDA action level in the feeds of dairy cattle (20 μg/kg). However, it represents a typical natural level of AFB1 in corn samples [23] and is within the range (20–100 µg/kg) used in previous studies [7,24].

**Figure 3:**
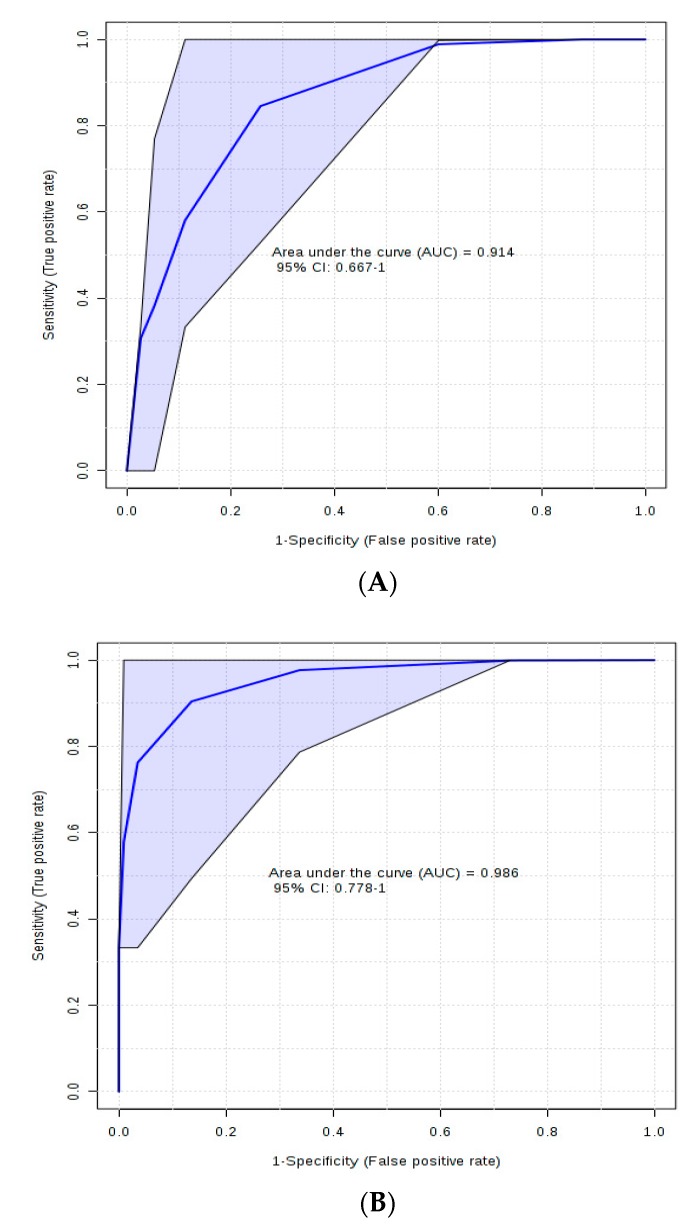
Receiver–operator characteristic curves of control vs. aflatoxin groups for (**A**) arginine and alanine and (**B**) arginine, alanine, methylhistidine, and citrulline.

The results of receiver–operator characteristic (ROC) analysis between C and T groups revealed five metabolites (arginine, alanine, methylhistidine, citrulline, and proline) with respective areas under the curve (AUC) of 0.88, 0.86, 0.86, 0.81, and 0.80. The utility of a biomarker is considered excellent at AUC = 0.9–1.0 and good at AUC = 0.8–0.9 [25]. Our previous study that applied 1H-NMR-based analysis revealed plasma acetic acid, arginine, ethanol, alanine, methylhistidine, and proline with AUC > 0.80 [12]; these results are similar except for ethanol and acetic acid, which were not quantified using DI/LC–MS/MS, and citrulline, which was not quantified in our previous study. This is an evidence that these metabolites, including ethanol and acetic acid reported in our companion paper, are good biomarkers of aflatoxin ingestion in dairy cows fed no sequestering agents. The combination of plasma concentrations of arginine and alanine gave a better ROC ability (AUC = 0.914; Figure 3a) compared with individual metabolites, whereas the combination of four metabolites (arginine, alanine, methylhistidine, and citrulline) with AUC = 0.986 was an excellent biomarker of aflatoxin ingestion in dairy cows fed no sequestering agents (Figure 3b). It is interesting to know that the use of multiple metabolites (arginine, alanine, methylhistidine, and citrulline) gave a better sensitivity and specificity to serve as potential biomarkers of aflatoxin ingestion in dairy cows fed no sequestering agents because it is likely that no single biomarker, as reported in our previous study using ^1^H-NMR, will accurately predict a disease condition. However, the optimum range of plasma concentrations of these metabolites in healthy lactating dairy cows has to be established across different feeding regimens for them to be useful as biomarkers of aflatoxin ingestion.

Dietary treatment with CL had no effects on any of the metabolites relative to the control, but increased (*p* < 0.05) concentrations of alanine, leucine, and arginine, and tended to increase (*p* = 0.07) that of citrulline relative to T. This indicates that dietary supplementation of clay prevented the negative effects of AFB1. The gastrointestinal tract (GIT) is the major route for entry of aflatoxins into the bloodstream, where they are distributed to various tissues and liver. Clay is effective at ameliorating the negative effects of aflatoxins on the health and performance of animals by binding with aflatoxin in the GIT, thereby, preventing it from being absorbed and getting to the liver, where it causes increased stress of the liver cells due to toxic load [26]. Several studies have reported that clay supplementation reduced transfer of aflatoxins into animal products, improving liver health and immune response in animals exposed to aflatoxin [6,16].

Dietary treatment with YEA resulted in higher concentrations of aspartic acid (0.09) and lysine (*p* = 0.05) relative to the control, and the highest plasma concentrations of alanine, valine, proline, threonine, leucine, isoleucine, glutamic acid, phenylalanine, and arginine compared with other treatments. This confirms that feeding SCFP with clay is better than clay alone at improving the health and metabolic status of dairy cows exposed to aflatoxins. This explains the results of our companion paper [6] that reported that dietary supplementation of clay and SCFP was more effective than clay alone at maintaining the milk yield of dairy cows during aflatoxin challenge. Several studies have shown that dietary supplementation with SCFP has the potential to optimize the health and performance of cattle, especially during inflammatory stress. However, the effectiveness of SCFP at augmenting inflammatory stress response to aflatoxin exposure has been inconsistent [4]. It is possible that observed effects of YEA in this study are due to the synergistic effects of clay and SCFP. Clay supplementation plays the role of adsorbing aflatoxins in the gut, thereby allowing maximal effects of SCFP at improving the nutritional status of the cows. This explains the increased milk production by cows fed YEA relative to T in our companion study [6]. Supplementation of SCFP has been shown to improve gut health by stabilizing rumen pH and increasing microbial N yield; resulting in increased flow and quality of amino acids to the duodenum for intestinal absorption [27,28]. In addition, the SCFP used in this study is fortified with nutritional metabolites such as amino acids, B vitamins, nucleotides, lipids, and organic acids that may have contributed to improved metabolic status of dairy cows in this study [29].

In summary, this study gives a comprehensive insight into plasma metabolomics profile in response to aflatoxin challenge with or without sequestering agents. Aflatoxin challenge reduced plasma concentrations of some amino acids. Clay supplementation prevented the effects of AFB1, while supplementation of both clay and SCFP improved the metabolic status of the cows during aflatoxin exposure. The combination of arginine, alanine, citrulline, and leucine had sufficient sensitivity and specificity to serve as candidate biomarkers of aflatoxin ingestion in dairy cows fed no sequestering agents.

## 3. Materials and Methods

This study was part of a larger project designed to evaluate the effects of supplementing clay with or without SCFP on health and performance of dairy cows challenged with AFB1 [6]. The University of Florida Institutional Animal Care and Use Committee approved all experimental procedures used in this study. Details about cows and feeding have been reported previously [6,12]. Briefly, eight lactating multiparous Holstein cows (64 ± 11 days in milk) were randomly assigned to one of four treatment sequences in a balanced 4 × 4 Latin square design with two replicate squares, four 33 day periods, and a 5 day washout interval between periods. Treatments were (1) control (diet with no additives), (2) toxin (T; basal diet + 1725 µg of AFB1/head per day), (3) toxin with bentonite clay (CL; 200 g/head per day; Astra-Ben-20, Prince Agri Products Inc., Quincy, IL, USA), and (4) CL plus SCFP (YEA; 35 g of SCFP/head per day; Diamond V, Cedar Rapids, IA, USA). The basal diet was formulated and fed as a total mixed ration (TMR), to meet the nutrient requirements of dairy cows producing 30 kg/d or more of milk [30]. The basal diet contained 55.6% concentrate mix, 36.1% corn silage, and 8.3% alfalfa hay on a dry matter basis. Oral dose of 1725 µg of AFB1 was administered to each cow in treatments T, CL, and YEA from days 26 to 30 before the morning feeding to give a dietary concentration of 75 µg/kg based on estimated daily DMI of 23 kg/d. The sequestering agents were top-dressed on the respective TMR from days 1 to 33 of each period. Blood samples (15 mL) were obtained from the coccygeal vessels into vacutainer tubes containing sodium heparin anticoagulant before the morning feeding on day 30 of each experimental period. Plasma samples were prepared from the blood by centrifugation at 2500× *g* for 20 min at 4 °C, initially stored at −20 °C and then at −80 °C until LC-MS/MS analysis was done.

Plasma samples (20 µL) were analyzed using a commercial kit that uses direct injection and tandem mass spectrometry (DI–MS/MS) coupled with a reverse-phase liquid chromatography and tandem mass spectrometry (LC–MS/MS). The kit assay (AbsoluteIDQ p180; Biocrates Life Sci., Innsbruck, Austria), used with an ABI 4000 Q-Trap mass spectrometer (Applied Biosystems/MDS Sciex, Foster City, CA, USA), enables the quantification of 145 metabolites in as little of 10 µL of biofluid. Detailed information and description of the method has been reported elsewhere [14,31]. Mass spectrometric analysis was performed using an API4000 Qtrap^®^ tandem mass spectrometry instrument (Applied Biosystems/MDS Analytical Technologies, Foster City, CA, USA) that is equipped with a solvent delivery system. Delivery of the samples to the mass spectrometer was done using an LC method, followed by a DI method.

## 4. Data and Statistical Analysis

Metabolite data were subjected to multivariate analysis using Metaboanalyst 4.0 software (www.metaboanalyst.ca) [24]. Prior to multivariate analysis, data were log-transformed and pareto-scaled. Partial least squares discriminant analysis (PLS-DA) was used to visualize differences between the control group and each of T, CL, and YEA groups. Based on the PLS-DA model, the metabolites that were important in discriminating cows in T, CL, and YEA from control group were ranked using variable importance in projection (VIP). Metabolites with VIP values ≥1 were considered powerful group discriminators [32]. Metabolites with VIP values ≥1 were analyzed using the GLIMMIX procedure of SAS version 9.4 (SAS Institute Inc., Cary, NC, USA, 2013). The model used for the analysis included the fixed effects of treatment, period, and random effects of cow and square, and their interactions. Significance was declared at *p* ≤ 0.05, and tendency was declared at 0.10 ≥ *p* > 0.05. Biomarker profiles for aflatoxin ingestion in dairy cows fed no sequestering agents were determined using receiver–operator characteristic (ROC) curves, as calculated by the ROCCET web server [25]. Area under the curve (AUC), a value that combines sensitivity and specificity for a diagnostic test, was used to evaluate the utility of the biomarkers [25]. 

## Figures and Tables

**Table 1:** The concentrations (µM) of plasma metabolites that were affected in dairy cows fed aflatoxin B_1_ with or without clay and SCFP ^1^-based sequestering agents.

Item	Treatment ^2^				SEM	*p*-Value
	Control	T	CL	YEA		
Alanine	337 ^b^	284 ^c^	334 ^b^	462 ^a^	18.1	0.01
Valine	331 ^y^	312 ^y^	325 ^y^	387 ^x^	14.5	0.06
Proline	143 ^b^	127 ^b^	137 ^b^	194 ^a^	7.47	0.01
Threonine	152 ^b^	136 ^b^	140 ^b^	172 ^a^	7.82	0.01
Leucine	281 ^b^	227 ^b^	284 ^b^	362 ^a^	14.2	0.01
Isoleucine	129 ^b^	131 ^b^	128 ^b^	148 ^a^	6.30	0.03
Aspartic acid	23.5 ^y^	26.9 ^xy^	29.5 ^xy^	43.5 ^x^	5.62	0.09
Glutamic acid	236 ^b^	219 ^b^	206 ^b^	305 ^a^	9.95	0.01
Arginine	128 ^b^	96 ^c^	131 ^b^	159 ^a^	10.4	0.02
Phenylalanine	68.4 ^b^	71.1 ^b^	69.4 ^b^	83.1 ^a^	4.37	0.05
Citrulline	73.6 ^xy^	62.6 ^y^	81.0 ^x^	71.6 ^xy^	4.81	0.07
Sarcosine	1.83 ^xy^	1.74 ^xy^	1.50 ^y^	1.95 ^x^	0.12	0.06
Lysine	159 ^b^	173 ^ab^	170 ^ab^	194 ^a^	9.05	0.05

^1^*Saccharomyces cerevisiae* fermentation product–based sequestering agent (Diamond V, Cedar Rapids, IA), ^2^ T = control diet + aflatoxin B_1_ (AFB1, 1725 μg/d); CL = T + 200 g/d of sodium bentonite clay; YEA = CL + 35 g/d of *Saccharomyces cerevisiae* fermentation product (SCFP). ^a,b,c^ Within a row, treatment means with different superscripts differ (*p* ≤ 0.05). ^x,y^ Within a row, treatment means with different superscripts tend to differ, 0.05 < *p* ≤ 0.10.

## Supplementary Materials

The following are available online at https://www.mdpi.com/2072-6651/11/12/693/s1, Table S1: Average concentrations (µm) of the identified metabolites.
